# A Novel MSCRAMM Subfamily in Coagulase Negative Staphylococcal Species

**DOI:** 10.3389/fmicb.2016.00540

**Published:** 2016-04-29

**Authors:** Srishtee Arora, Anne-Catrin Uhlemann, Franklin D. Lowy, Magnus Hook

**Affiliations:** ^1^Center for Infectious and Inflammatory Diseases, Institute of Biosciences and Technology, Texas A&M University Health Science Center, HoustonTX, USA; ^2^Division of Infectious Diseases, Department of Medicine, College of Physicians and Surgeons, Columbia University in the City of New York, New YorkNY, USA

**Keywords:** cell wall anchored proteins, MSCRAMM, *Staphylococcus epidermidis*, coagulase negative staphylococci, structural homologs, N-terminal repeats

## Abstract

Coagulase negative staphylococci (CoNS) are important opportunistic pathogens. *Staphylococcus epidermidis*, a coagulase negative staphylococcus, is the third leading cause of nosocomial infections in the US. Surface proteins like Microbial Surface Components Recognizing Adhesive Matrix Molecules (MSCRAMMs) are major virulence factors of pathogenic gram positive bacteria. Here, we identified a new chimeric protein in *S. epidermidis*, that we call SesJ. SesJ represents a prototype of a new subfamily of MSCRAMMs. Structural predictions show that SesJ has structural features characteristic of a MSCRAMM along with a N-terminal repeat region and an aspartic acid containing C-terminal repeat region, features that have not been previously observed in staphylococcal MSCRAMMs but have been found in other surface proteins from gram positive bacteria. We identified and analyzed structural homologs of SesJ in three other CoNS. These homologs of SesJ have an identical structural organization but varying sequence identities within the domains. Using flow cytometry, we also show that SesJ is expressed constitutively on the surface of a representative *S. epidermidis* strain, from early exponential to stationary growth phase. Thus, SesJ is positioned to interact with protein targets in the environment and plays a role in *S. epidermidis* virulence.

## Introduction

Coagulase negative staphylococci (CoNS), which colonize human skin and mucus membranes, are recognized as important opportunistic pathogens. *Staphylococcus epidermidis* and *Staphylococcus haemolyticus* are the two most prevalent CoNS species responsible for causing a significant proportion of device-related, health care-associated infections and infections in preterm newborns (Becker et al., 2014). *S. epidermidis* alone is the third leading cause of nosocomial infections in the US. *Staphylococcus saprophyticus* is the second most common cause of uncomplicated urinary tract infections in sexually active women between the age of 18 and 35 years (Wallmark et al., 1978; Latham et al., 1983). Recent studies have indicated that *Staphylococcus capitis* can cause late onset sepsis in very low birth weight infants in the neonatal intensive care unit setting (Van Der Zwet et al., 2002; Gras-Le Guen et al., 2007) and prosthetic valve endocarditis in adults (Petti et al., 2008).

Bacterial surface proteins such as the cell wall-anchored (CWA) proteins have been identified as important virulence factors among gram positive bacterial pathogens and play key roles in microbial adherence to host tissues, evasion of host defense systems and biofilm formation (Foster and Hook, 1998; Foster et al., 2014). For example, in *S. epidermidis* the CWA proteins Biofilm associated protein (Bap) (Tormo et al., 2005), Accumulation associated protein (Aap) (Schaeffer et al., 2015), Serine-aspartate dipeptide repeat protein F (SdrF) (Arrecubieta et al., 2009), SdrG (Pei and Flock, 2001) and Extracellular matrix-binding protein (Embp) (Christner et al., 2010) all can participate in biofilm formation either by mediating bacterial attachment to matrix proteins or intercellular aggregation. Furthermore antibodies to the *Staphylococcus epidermidis* surface protein C (SesC) inhibit biofilm formation, but a molecular function for SesC in biofilm formation has not yet been determined (Shahrooei et al., 2009, 2012).

The CWA proteins of gram positive bacteria are often modular proteins composed of various interlinked domains. These proteins can be further divided into families [e.g., Microbial Surface Components Recognizing Adhesive Matrix Molecules (MSCRAMMs) and Serine Rich Repeat Proteins (SRRPs)] based on the presence of common characteristic domains (Lizcano et al., 2012; Foster et al., 2014). A defining feature of the MSCRAMM family is the presence of two tandemly linked IgG-like folded domains, which can engage in ligand binding by the dock, lock and latch (DLL) mechanism (Ponnuraj et al., 2003; Bowden et al., 2008). A subfamily of MSCRAMMs contains a repeat (R) region composed of serine-aspartate di-peptide repeats (Sdr), which defines the Sdr protein subfamily. In *S. aureus* this subfamily includes Clumping factor protein A (ClfA), ClfB, SdrC, SdrD and SdrE (Foster and Hook, 1998; Foster et al., 2014). There are two members of the Sdr subfamily on the surface of *S. epidermidis*; SdrG and SdrF (Bowden et al., 2005). SdrG binds to human fibrinogen (Ponnuraj et al., 2003). SdrF binds to Type I Collagen and is involved in the initiation of left ventricular assist device driveline infections (Arrecubieta et al., 2009).

The SRRP family of CWA proteins is defined by the presence of a serine repeat region (SRR), which is similar to the R region of Sdr proteins in that it contains serine dipeptide repeats but with either alanine, valine or threonine as the partner residue. SRRPs have a N-terminal signal sequence, at most two unique non-repeat (NR) regions, two SRRs flanking the NR region(s) and motifs needed for cell wall anchoring at the C-terminus (Lizcano et al., 2012). Based on the crystal structures of NRs of two SRRPs; Fap1 from *S. parasanguinis* and GspB from *S. gordonii*, the NR region can be further subdivided into different domains. Both proteins contain one IgG-like folded subdomain in the NR region (Ramboarina et al., 2010; Pyburn et al., 2011). The SRRPs are common in streptococci but also have been found in *S. aureus* (SraP in strain N315, Siboo et al., 2005), *S. haemolyticus* (SH0326 in strain JCSC1435, Takeuchi et al., 2005) and *S. epidermidis* (SE2249 in strain ATCC 12228, Zhou and Wu, 2009).

Although the pathogenic mechanisms of *S. epidermidis* are attracting more attention, the possible roles of CWA proteins in these infections need to be further examined. Furthermore, CWA proteins on other CoNS are even less well characterized. We here report on the discovery of a previously unknown CWA *S. epidermidis* protein that we demonstrate is a prototype of a novel subfamily of CoNS MSCRAMMs.

## Materials and Methods

### Bacterial Strains and Growth Conditions

*S. epidermidis* strains were routinely grown in Tryptic Soy Broth (TSB) medium overnight at 37°C at 200 rpm. Growth curves were generated by inoculating fresh TSB media with overnight inoculum to a starting OD600 of 0.03, followed by incubation at 37°C at 200 rpm. *S. epidermidis* strain 3094 and 2111 are clinical isolates obtained from patients with left ventricular device driveline infections. *S. epidermidis* 3094 was used as a source for cloned constructs. *Escherichia coli* strains XL1Blue and BL21 Acella^TM^ were grown in Luria-Bertani (Sigma) medium with appropriate antibiotics at 37°C.

### Identification of SesJ Structural Homologs

Bacterial genome sequences in the NCBI database were searched for proteins with similarity to the SesJ protein using BLAST. New proteins were only accepted if they contained N-terminal Repeats (NTRs) at the N-terminus and dipeptide repeats at the C-terminus. Search results were further analyzed to select for proteins that contained a N-terminus signal sequence, A-region, B repeats, a LPXTG motif, transmembrane domain followed by a positively charged C-terminal amino acid sequence. Online bioinformatics tools were used to characterize protein sequences from the BLAST search. The repeat domains were identified visually and using the Internal Repeat Finder^1^ algorithm. Protein secondary and tertiary structure was predicted using Protein Homology/analogY Recognition Engine V 2.0 (Phyre^2^)^2^. Sequence alignment and protein identity was calculated with Clustal Omega algorithm program^3^. N-terminus signal sequence was predicted using SignalP 4.1 server^4^. Hydrophobic transmembrane domain was predicted using TMHMM Server v. 2.0^5^.

### Construction and Purification of Histidine-Tagged Fusion Proteins

rSesJ_258-637_ protein was expressed with hexahistidine tag at the N-termini using the expression vector pQE30 (Qiagen). PCR primers used were SdrS N2N3 fwd (TAG**GGATCC**CCAGAGGTTGATTCCGAAGTATTAG) and SdrSA rev (TAG**GTCGAC**CTAAAGTTTTTCATTGCCAGTAGCAAC). Genomic DNA from *S. epidermidis* strain 3094 was isolated using UltraClean^®^ Microbial DNA Isolation kit (MO BIO Laboratories, Inc.). Expression cultures were induced with IPTG and protein was purified using nickel-affinity chromatography and anion-exchange chromatography as described previously (Davis et al., 2001). Binding buffer (20 mM sodium phosphate, 1 M NaCl, pH 8.0), wash buffer (20 mM sodium phosphate, 1 M NaCl, 25 mM Immidazole) and elution buffer (20 mM sodium phosphate, 1 M NaCl, 500 mM Immidazole, pH 8.0) were used for nickel-affinity chromatography. Binding buffer (20 mM Tris, pH 8.0), wash buffer (20 mM Tris, 50 mM NaCl, pH 8.0) and elution buffer (20 mM Tris, 500 mM NaCl, pH 8.0) were used for anion-exchange chromatography.

### Flow Cytometry

To determine surface expression of SesJ by flow cytometry, bacteria were grown for 4 h in TSB broth from an inoculum with an OD600 of 0.03. Collected and washed cells were labeled with preimmune or SesJ antiserum followed by Alexa Fluor 488 conjugated goat anti-rabbit IgG as described previously. Cells were fixed with 3% paraformaldehyde in PBS and analyzed with BD Accuri^TM^ C6 cytometer. Polyclonal antibodies against rSesJ_258-637_ were raised in rabbit (Rockland Immunochemicals Inc.). Pre-immune sera was tested for reactivity to SesJ using ELISA before selecting an animal for antibody production.

### Sequence Logo

Sequence logo was generated using frequency plot at WebLogo online program^6^ (Schneider and Stephens, 1990; Crooks et al., 2004). Custom color scheme as described in the figure legend was used to generate the graph.

### PCR Screening

Ninety five coagulase-negative Staphylococcus colonizing and infections isolates were used to determine the distribution of the *sesJ* gene, including 64 isolates from a study on the epidemiology of *S. epidermidis* colonization and infection in left ventricular assist device individuals (Gordon et al., 2013). DNA from picked colonies was amplified using primers SdrS-F 5′-GAGCACAGACAATTCGACTTCAAATC and SdrS-R TCAGCATATTCCGGCATATCTACTG and PCR products were sequenced for confirmation.

## Results

### Sequence Analysis of the SesJ Protein

**FIGURE 1:**
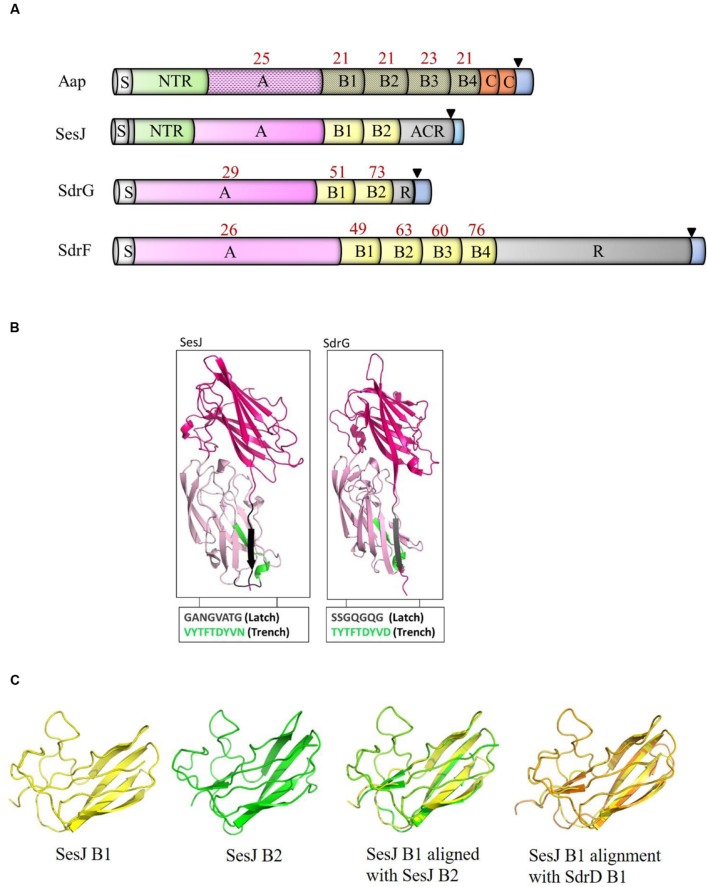
**Comparison of SesJ with other structurally related proteins.** **(A)** Schematic representation of *S. epidermidis* surface proteins SesJ, SdrG, SdrF and Aap. Cartoons show the relative position of NTRs (SesJ and Aap) to other domains. The number in red above the individual domains represents its identity to the corresponding SesJ domain. The signal sequence is shown in white, NTRs in green, A-region in pink, B repeats in yellow, R region and ACR region in gray, collagen triple helix in orange, and cell wall spanning region and cytoplasmic tail in blue. LPXTG motif is shown using a black triangle. **(B)** Modeled 3D structure of the A-region of SesJ comprising two IgG-like folded domains compared to the crystal structure of SdrG_N2N3_. **(C)** Structure predictions of B1 (yellow), B2 (green) repeat of SesJ, along with an overlay of SesJ B1 (yellow) and B2 (green), and SesJ B1 (yellow) with SdrD B1 (orange) repeat.

While examining the sequence variation of SdrG in published *S. epidermidis* genomes, we discovered a gene encoding a SdrG-like but clearly distinct CWA protein that we have called SesJ (GenBank accession number: KU935462) according to the established nomenclature. The deduced full length SesJ protein in strain 3094 is 1047 amino acids long (**Figure 1A**). The predicted molecular mass of the mature SesJ protein after cleavage of the signal sequence and processing by sortase is 105.82 kDa. Amino acid sequence analysis of the full length protein revealed that SesJ has 41 and 40% sequence identity to SdrG (GenBank accession number: AAF72510.1) and SdrF (GenBank accession number: AAF72509.1), respectively. SesJ is a multidomain protein that contains, starting from the N-terminus, a 44 amino acid long signal sequence, a NTR region, an A-region, two B repeats, an aspartic acid containing repeat (ACR) region and typical cell wall anchoring sequences such as a LPXTG motif, a hydrophobic membrane spanning region and a short cytoplasmic positively charged tail (**Figure 1A**).

### SesJ is a Novel Chimeric MSCRAMM

MSCRAMMs bind to their ligands through the DLL mechanism (O’Connell, 2003; Ponnuraj et al., 2003; Bowden et al., 2008). This binding mechanism involves characteristic structural features in the MSCRAMM A-region including two adjacent IgG-like folded domains where a conserved TYTFTDYVD-like motif is present at the “back” of the latching trench in the first domain in the tandem and a latch sequence at the C-terminal extension of the second domain. A latch sequence is not a conserved sequence of amino acids but consists of small uncharged, polar and non-polar residues (Ponnuraj et al., 2003). Tertiary structure prediction using the PHYRE^2^ fold recognition server (Kelley et al., 2015) indicated that residues 269–634 in the A-region of SesJ are highly likely to adopt two IgG-like folds (100% confidence level). The predicted structure of the two IgG-like domains in SesJ is very similar to the crystal structure determined for the N2N3 domain of SdrG (Ponnuraj et al., 2003) (**Figure 1B**). This SesJ segment furthermore contains the sequence, VYTFTDYVN at the expected positions for a latching trench in the first domain of the predicted IgG-like tandem. In addition, a putative latch sequence GANGVATG is present in the extension of the second IgG-like folded domain of SesJ. The presence of a latch sequence and a conserved TYTFTDYVD-like motif in the N2N3 subdomain indicates that SesJ could bind a ligand peptide by the DLL mechanism (**Table 1**). In summary, the A-region of SesJ contains the characteristic IgG-like folded tandem of a MSCRAMM.

**Table 1:** Conserved motifs in the A-region of SesJ structural homologs.

Protein	Trench motif^∗^	Latching sequence
SesJ	VYTFTDYVN	GANGVATG
ScsJ	VYTFTDYVN	GANGVATG
ShsJ	KYTFTDYVN	GANGIAQG
SdrI	TYTFTNYVD	GSSTAQG


*^∗^Residues in indicate conserved residues amongst the proteins.*

As a result of the unusually short N1 region that connects the N2N3 tandem to the preceding NTR region, the A-region of SesJ (378 amino acids) is smaller compared to the A-regions of SdrG (546 amino acid) and SdrF (624 amino acid). Sequence comparison shows that the amino acid sequence of the SesJ A-region is 29 and 26% identical to the comparable A-region segments of SdrG and SdrF, respectively (**Figure 1A**). Similar levels of sequence identity are also seen between the A-regions of SesJ and the various *S. aureus* MSCRAMMs.

Staphylococcal MSCRAMMs do not usually contain a NTR domain, which is found in SesJ between the signal sequence and the A-region (**Figure 1A**). The NTR domain of SesJ is composed of a 15 amino acid long sequence repeated 10 – 13 times depending on the strain (**Table 2**). A NTR domain is also found in one other CWA protein from *S. epidermidis*; Aap, where it is composed of a variable number of a 16 amino acid long sequence (**Table 2**). There is a low level of sequence identity throughout the Aap (GenBank accession number: AAW53239.1) and SesJ proteins (**Figure 1A**). The NTRs of the two proteins show intriguing similarities (further discussed below) but structure prediction show that the A-region of Aap is likely to adopt a lectin type fold, which is distinctly different from that of the characteristic MSCRAMM tandem (Bowden et al., 2005).

**Table 2:** Summary of NTRs of SesJ structural homologs.

Protein	Sequence of NTR^∗^	Number/Length of repeat sequence
SesJ	– – – – –EAPSKEEAPSNEATN	13/15
ScsJ	– – – – –EEPSKEEATSKEVTN	8/15
ShsJ	– – – – –EQASTEEKADTT– – –	24/12
SdrI	– – – – –EPATKEEAATTE– – –	23/12
Aap	EAPQSEPTKTEEGSNA– – – –	12/16


*^∗^Residues in indicate conserved residues amongst the proteins.*

SesJ contains two B repeats that are composed of 110 – 111 amino acids, similar in length to B repeats of the Sdr subfamily of staphylococcal MSCRAMMs. The SesJ B repeats show 63–69% sequence identity to B repeats of SdrG and SdrF (**Figure 1A**). Similar to SdrG and SdrF, the B repeats of SesJ also harbor predicted Ca^2+^ binding sites (Josefsson et al., 1998). The predicted structures of SesJ B1 and B2 are shown (**Figure 1C**) and these are similar to the crystal structure determined for the *S. aureus* SdrD B1 (Wang et al., 2013). On the other hand the crystal structure of the Aap B domain, which consists of two subdomain G5 and E (Gruszka et al., 2012; Conrady et al., 2013) is very different from those predicted for the SesJ B domains.

The ACR region of SesJ is distinct from the SD repeated dipeptide characteristic of the Sdr protein subfamily of staphylococcal MSCRAMMs. A 20 amino acid long motif [SESTSESDSESHSDSES(H/D)SD] is repeated in the ACR region of SesJ. This type of arrangement is similar to the SRRs of SRRPs, which also are composed of longer motifs, e.g., SAS(T/E)SASTSASV in *S. gordonii* Challis (Lizcano et al., 2012). We propose to name this segment ACRs since structural homologs of SesJ found in other CoNS species (see below) contain similar C-terminal repeat motifs where an aspartic acid (rather than serine) is the conserved residue.

These comparative analyses reveal that SesJ is a new unique chimeric protein that has acquired structural motifs from other families of CWA proteins. The NTR are related to the corresponding segment of Aap the IgG-like folded domains have all the features of a MSCRAMM and the ACR region resembles the SRR of streptococcal SRRPs.

### SesJ is Expressed on the Surface of *S. epidermidis*

**FIGURE 2:**
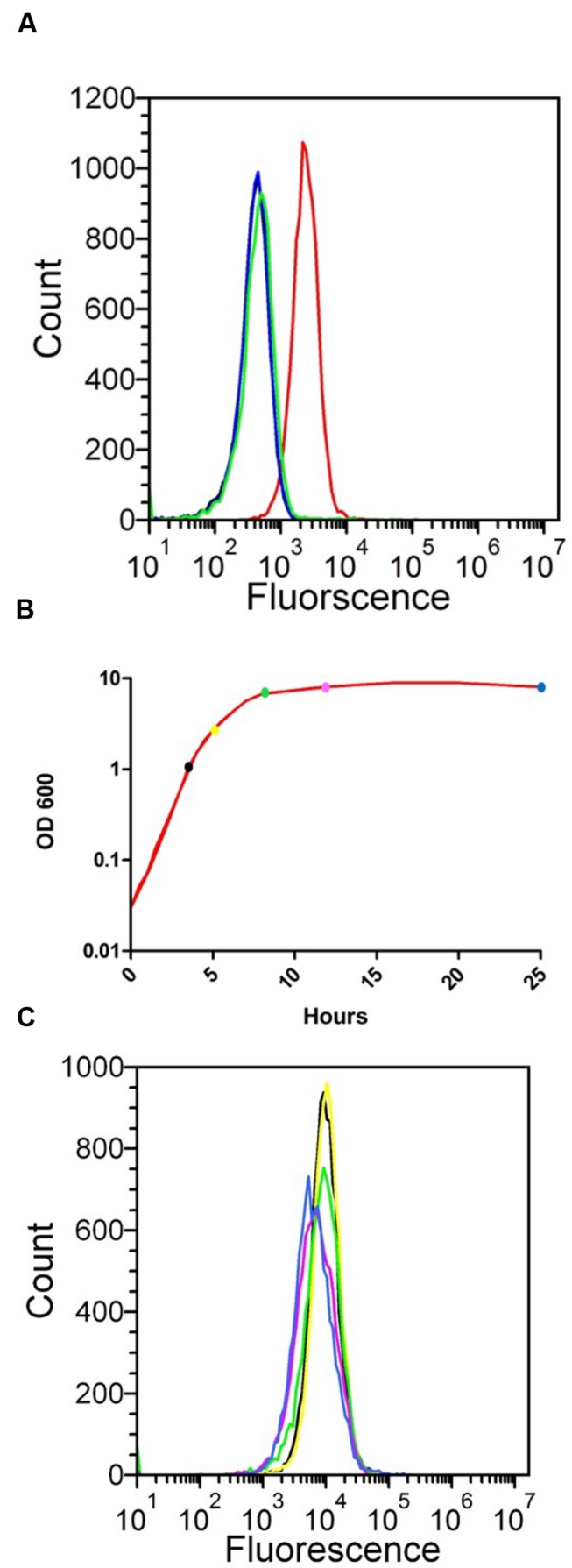
**Detection of SesJ expression on the surface of *S. epidermidis* by flow cytometry.** **(A)** Comparison of SesJ expression on the surface of *sesj*^+^ *S. epidermidis* 3094 (solid red line), *sesj*^-^ *S. epidermidis* 2111 (solid green line), *S. epidermidis* 3094 treated with pre bleed serum and secondary antibody (solid black line) and *S. epidermidis* treated with secondary antibody only (solid blue line). **(B)** Growth curve of *sesj*^+^ *S. epidermidis* strain 3094 in red line. Three hours is represented by black solid circle, 5 h by yellow solid circle, 8 h by green solid circle, 12 h by magenta solid circle and 25 h by blue solid circle. **(C)** Comparison of SesJ expression on the surface of *sesj*^+^ *S. epidermidis* strain 3094 at 3 h (solid black line), 5 hrs (solid yellow line), 8 h (solid green line), 12 h (solid magenta line), 25 h (solid blue line).

The deduced amino acid sequence of SesJ suggests that this protein could be expressed on the bacterial surface as a cell wall anchored protein. We verified by flow cytometry that SesJ in fact was expressed on the surface of *S. epidermidis* strain 3094 when bacteria were grown to mid exponential phase, 4 h (**Figure 2A**). *S. epidermidis* strain 2111, which does not have the *sesJ* gene, was used as a negative control to study expression of SesJ. The SesJ antisera did not cross react with any surface protein on the *S. epidermidis* 2111 strain. Similarly, pre-immune sera and secondary antibody alone did not bind to surface molecules on the *S. epidermidis* 3094 strain (**Figure 2A**). We next generated a growth curve (**Figure 2B**) and looked for expression of the protein at different time points during the growth curve. SesJ was constitutively expressed on the surface of *S. epidermidis* strain 3094 throughout the growth curve (**Figure 2C**).

### Structural Homologs to SesJ are Present in other CoNS

**FIGURE 3:**
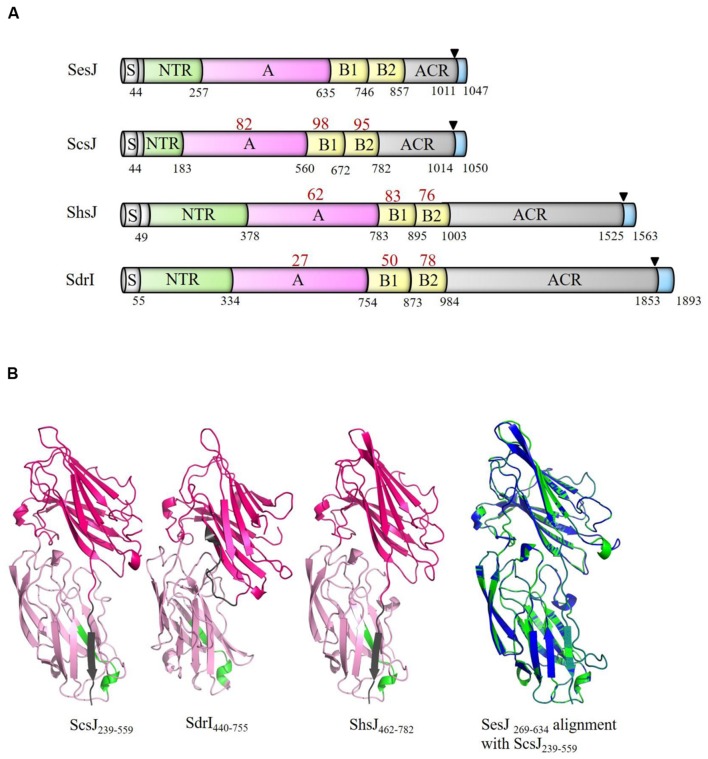
**SesJ structural homologs in other CoNS species.** **(A)** SesJ structural homologs have the same relative position of structural domains. The number in red below the individual domains represents its identity to the corresponding SesJ domain. Numbers in black represent the end of domain. Signal sequence is shown in white, NTRs in green, A-region in pink, B repeats in yellow, ACR region in gray, and cell wall spanning region and cytoplasmic tail in blue. LPXTG motif is shown using a black triangle. **(B)** Predicted structures of ScsJ_239-559_, SdrI_440-755_, ShsJ_462-782_ and overlay of SesJ_269-634_ (blue) with ScsJ_239-559_ (green).

We then searched for structural homologs in available sequenced genomes in the NCBI genome database for ORFs that contained NTRs and ACR region. Search results were further sorted for the presence of an A-region, B repeats and a LPXTG motif. We identified structural homologs of SesJ in *S. capitis, S. haemolyticus and S. saprophyticus* (**Figure 3A**). Two of these had not been previously identified and we have named these proteins ScsJ (*S. capitis* Strain CR01, GenBank accession number: WP_016898462.1) and ShsJ (*S. haemolyticus* strain JCSC1435, GenBank accession number: BAE03349.1). We found that the structural homolog in *S. saprophyticus* has already been reported and named SdrI (*S. saprophyticus* strain 7108, GenBank accession number: AAM90673.1) (Sakinc et al., 2006). All these SesJ structural homologs contain a NTR region, an A-region, two B repeats and an ACR region as well as the characteristic cell wall anchoring motifs. The predicted lengths of the proteins vary considerably from 1048 to 1893 residues due to variations in the NTR and ACR regions.

The A-regions of these proteins are similar in length and range from 378 amino acid to 420 amino acid (**Figure 3A**). Tertiary structure prediction using the PHYRE^2^ fold recognition server (Kelley et al., 2015) indicated that residues in the A-region of the identified structural homologs is highly likely to also adopt the MSCRAMM characteristic IgG-like folded tandems (100% confidence; **Figure 3B**). The conserved TYTFTDYVD-like motif is found in the first IgG-like domain and the extension of the second IgG-like domain contains a putative latch sequence (**Table 1**). The B repeats, which are of the Sdr protein types are similar in size (range from 108 amino acid – 120 amino acid) and contain putative Ca^2+^ binding sites.

SesJ is most similar to ScsJ from *S. captis* with sequence identity of 82 and 96% for the A-region and B repeats, respectively. The A-region of SesJ has about 62% identity with ShsJ A-region but less than 30% identity to that of SdrI. The B repeats are relatively well conserved in all identified members of this subfamily. B2 repeats are most conserved with over 75% identity whereas the B1 repeats show slightly more variations (up to 50%) (**Figure 3A**). This suggests that the A-region of these proteins may interact with different ligands.

The repeats in the ACR region of the proteins vary in amino acid composition and length of the repeat motif. ScsJ has the same residues in the ACR region as SesJ and a similar motif, i.e., SESESESHSDSESHSDSEST. ShsJ has a 12 amino acid repeat motif S(T/Q)SDSES(T/Q)SDSE which is shorter than repeat motifs in the ACR region of SesJ and ScsJ. SdrI has both serine-aspartic acid dipeptide and alanine-aspartic acid dipeptide repeats with a repeat motif SD_(1-2)_AD_(1-5)_.

### The NTRs Contain a Conserved Motif

**FIGURE 4:**
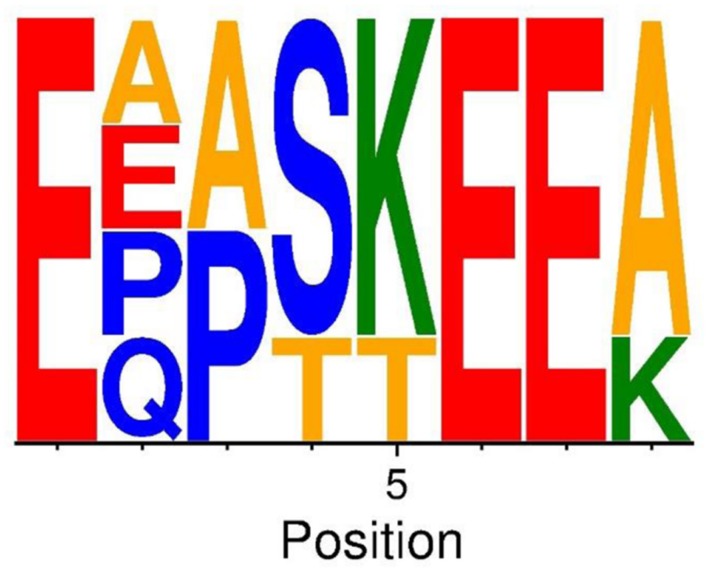
**Conserved motif in the NTRs of SesJ structural homologs.** Sequence logo of conserved 8 amino acid motif amongst all the structural homologs. Basic residues are colored in green, the acidic residues in red, the neutral ones in blue, the hydrophobic ones in yellow and remaining residues in orange.

The presence of NTRs defines this MSCRAMM subfamily but the number, length and sequence of the individual repeat units vary among the identified subfamily members (**Table 2**). The repeat units are rather long; 12–15 residues and are composed of mostly hydrophilic amino acids. Within NTRs of individual proteins the sequence in the first and last repeat units diverges somewhat from the sequence of repeats in the core of the NTRs. Despite the extensive variation among the sequences of different NTRs, an eight amino acid long conserved motif can be identified (**Figure 4**). Intriguingly the 16 amino acid long repeat unit in the NTR of Aap also contain a variant of the core motif [E^∗∗∗^(K/T)EE] found in NTRs of SesJ homologs (**Table 2**).

### Prevelence of SesJ Structural Homologs in CoNS

We examined a total of 95 *S. epidermidis* isolates to determine the prevalence of the *sesJ* gene. Seventeen isolates (18%) were positive and the *sesJ* gene was present in both colonizing and infectious isolates. Furthermore, 4/26 (15%) *S. capitis* bloodstream isolates were positive for the *scsJ* gene. These frequencies are similar to the reported presence of *sdrI* (11%) in *S. saprophyticus* UTI samples (Sakinc et al., 2006). Thus, members of the new subfamily of MSCRAMMs are present in 11–18% of the examined CoNS isolates.

## Discussion

Pan genome sequence analysis has revealed the plasticity of the CoNS species genomes. (Takeuchi et al., 2005; Conlan et al., 2012). These include CoNS genome changes by the addition of new genes, which can alter the virulence potential of the different subspecies. Sequencing of more genomes aids in the identification of new virulence factors. In this study, we identified and characterized a novel MSCRAMM SesJ, which is a chimeric protein with structural features characteristic of staphylococcal MSCRAMMs. The NTR and ACR regions of SesJ are not found in other MSCRAMMs but are related to similar domains in Aap and SRRPs, respectively. Furthermore, SesJ is a prototype of a new subfamily of MSCRAMMs. This newly identified family consists of the structural homologs SesJ, ScsJ, ShsJ and SdrI, present in ∼11–18% of *S. epidermidis*, *S, capitis*, *S. haemolyticus*, and *S. saprophyticus*, respectively. We also verified that SesJ is expressed constitutively on the surface of *S. epidermidis.*

Protein families based on structural homology have been reported before. Many gram positive bacteria express members of a family of structurally related collagen binding proteins where CNA of *S. aureus* is a prototype (Patti et al., 1994). Similarly, the SRRPs constitute a family of structurally related proteins present in different streptococcal and staphylococcal species (Lizcano et al., 2012). Structural homologs of Bap are present in *S. epidermidis*, *S. chromogenes*, *S. hyicus*, *S. xylosus*, *S. simulans* as well as in *S. aureus* (Tormo et al., 2005).

The *S. aureus* MSCRAMMs have been studied in most detail. Although these proteins all have sequence features required for the structural organization of MSCRAMM their overall sequences can vary significantly (Foster and Hook, 1998; Foster et al., 2014). These sequence variations allow individual MSCRAMMs to have different functions, target different ligands or different sequences in the same ligand. For example, fibrinogen is a ligand for many of the *S. aureus* MSCRAMMs but ClfA, ClfB and Bbp bind to different sites in the fibrinogen molecule. The sequence variations seen among the SesJ like proteins in different CoNS suggests that these proteins do not necessarily target the same ligand or even have the same function in the different species.

The C-terminal repeat regions of Sdr proteins and SRRPs are hypothesized to extend the ligand binding domain, i.e., the A-region of Sdr proteins and NR region of SRRPs, away from the cell surface to prevent obstruction of ligand binding by the cell wall (Hartford et al., 1997; Lizcano et al., 2012). The C-terminal repeat regions of Sdr proteins and SRRPs are often glycosylated. SRRPs are encoded at loci harboring genes for two glycosyltransferases. Sdr proteins also have two glycosyl transferase *sdgA*, *sdgB* gene encoded downstream of SdrC/D/E proteins (Hazenbos et al., 2013). Likewise, two genes predicted to encode glycosyl transferases are located immediately upstream of the *sesJ* gene (not shown) and we therefore speculate that SesJ is glycosylated. These observations indicate that SesJ is subjected to similar post translational modifications as the Sdr proteins and the SRRPs.

Cell wall anchored proteins of gram positive bacteria contain a characteristic C-terminal region where the LPXTG motif is recognized by the transpeptidase sortase, which covalently attaches the protein to the peptidoglycan on the surface of the organism. Flow cytometry confirmed that SesJ is present on the surface of *S. epidermidis*. Furthermore, the protein is present from early logarithmic to late stationary phase in strain 3094. In *S. aureus*, expression levels of MSCRAMMs can vary during the growth phase. For example, expression of FnbpA on the surface of *S. aureus* Newman increases during late logarithmic phase and decreases in the late stationary phase. In contrast, ClfB increases during early logarithmic phase and disappears at early stationary phase (Ythier et al., 2012). The expression of SdrG on the surface of *S. epidermidis* strain 0-47 was not induced under the tested *in vitro* growth conditions but increased over 30 fold in 3 h in a murine infection model (Sellman et al., 2008). Thus the expression of Sdr proteins on staphylococci can differ, depending upon the nature of the protein and the environment, and these variations may relate to the role of the proteins in the pathogenic mechanism of the organism. Additional studies of the expression of SesJ in different strains and under different conditions are warranted and can provide clues on the role of the encoded protein in the life of *S. epidermidis*.

The A-region is the primary ligand binding region of MSCRAMMs, which often uses the DLL mechanism to engage the targets. SdrG_N2N3_ binds to a specific sequence in the N-terminal section in the β-chain of human fibrinogen by this mechanism (Davis et al., 2001; Bowden et al., 2008). However, B repeats may also engage in ligand binding. For example SdrF binds Type I collagen via its B repeats in a temperature dependent manner (Di Poto et al., 2015). MSCRAMMs bind to host proteins and can mediate bacterial adhesion, evade immune response as well as participate in biofilm formation. Both SdrG and SdrF initiate biofilm formation by binding to human fibrinogen and Type I collagen, respectively (Ponnuraj et al., 2003; Arrecubieta et al., 2009). Aap participates in both the initial attachment phase, where a region containing the NTRs and the A domain is required, and in the accumulation phase, where the B domains seem to be involved (Conrady et al., 2013; Conlon et al., 2014). SRRPs acts as adhesins and colonize host tissues by forming biofilms. SesJ’s similarity to MSCRAMMs and domains from proteins involved in biofilm formation, leads to a speculation that SesJ might be involved in biofilm formation and/or interact with host proteins. The interactions of SesJ and its possible role in *S. epidermidis* pathogenesis are currently under investigation.

## Author Contributions

SA, A-CU performed the experiments. SA, A-CU, FL, and MH planned the experiments. SA, A-CU, FL, and MH participated in the writing of the manuscript.

## Conflict of Interest Statement

The authors declare that the research was conducted in the absence of any commercial or financial relationships that could be construed as a potential conflict of interest.

## Footnotes

1 http://nihserver.mbi.ucla.edu/Repeats/

2 http://www.sbg.bio.ic.ac.uk/phyre2/html/page.cgi?id=index

3 http://www.ebi.ac.uk/Tools/msa/clustalo/

4 http://www.cbs.dtu.dk/services/SignalP/

5 http://www.cbs.dtu.dk/services/TMHMM/

6 http://weblogo.berkeley.edu/logo.cgi
